# New Medical Journal

**Published:** 1865-04

**Authors:** 


New Medical Journal.—We have the pleasure of announcing the appearance of "The New York Medical Journal," issued in good style, with an almost ostentatious display of collaborators. That it will be ably conducted and in every way worthy the confidence and support of the profession, there can be no doubt. It will be published the first of each month, and will be sustained by the talent of some of the most distinguished members of the profession in this country. We hope it is established upon a permanent basis.

